# Measurement of social and emotional wellbeing and mental health outcomes in first nations children: A systematic review

**DOI:** 10.1002/jcv2.70147

**Published:** 2026-07-17

**Authors:** Maddison O’Gradey‐Lee, Emma A. McDermott, Lizel‐Antoinette Bertie, Clinton Schultz, Jennifer L. Hudson

**Affiliations:** ^1^ The Black Dog Institute University of New South Wales Sydney New South Wales Australia; ^2^ School of Psychology University of New South Wales Sydney New South Wales Australia; ^3^ Psychiatry and Mental Health, School of Clinical Medicine University of New South Wales Sydney New South Wales Australia

**Keywords:** child, first nations, measurement, mental health, social and emotional wellbeing

## Abstract

**Background:**

There is a critical lack of reliable, high‐quality epidemiological data on mental health and/or social and emotional wellbeing (SEWB) outcomes for First Nations children, partly, due to the limited availability of culturally valid assessment tools. This review aims to assess the cultural validity of mental health and SEWB assessment tools used with First Nations children aged 4–12 years in Australia, and identify gaps, strengths, and opportunities for reform that enhance cultural safety, and self‐determination in assessment practices.

**Methods:**

A systematic search of five electronic databases (Web of Science, PubMed, PsycINFO, Informit, and CINAHL) identified English‐language studies (1980–2025) assessing mental health or SEWB in Aboriginal and/or Torres Strait Islander children aged 4–12 years. Data on assessment tools were extracted and their cultural validity analysed using the First Nations Cultural Validity Assessment Tool. Tools were classified, as bespoke, culturally adapted or generic, using the CBSPATSISP definitions.

**Results:**

This review examined the cultural validity across 10 studies, including 11 unique tools and 16 assessments of cultural validity. Three tools were bespoke, eight culturally adapted, and five generic. Over three quarters of tools used to assess SEWB or mental health in First Nations children had poor or limited cultural validity. Two bespoke SEWB tools were identified, although none specifically targeted mental health.

**Conclusion:**

Culturally valid assessment tools for First Nations children remain limited, with research predominantly relying on inappropriate measures. Improving the measurement of mental health and SEWB outcomes requires a shift towards First Nations‐led, co‐designed tools grounded in Indigenous knowledges, strengths, and cultural frameworks. The evidence base was limited by reliance on published academic sources, which may have missed community‐used assessment tools, a focus on cultural validity rather than broader study quality, and limited psychometric reporting for some First Nations‐specific measures.

**Trial Registration:**

PROSPERO (CRD42024542866). 13 May 2024. www.crd.york.ac.uk/PROSPERO/view/CRD42024542866.

## Key Points

**What's known?**

- There is a lack of high‐quality epidemiological data on mental health and social and emotional wellbeing (SEWB) in First Nations children.
- Available assessment tools are often developed for non‐Indigenous populations and may lack cultural validity.

**What's new?**

- This is the first systematic review to evaluate the cultural validity of SEWB and mental health measures for First Nations children using the First Nations Cultural Validity Assessment Tool (FN‐CVAT) framework.
- Most tools demonstrated poor or limited cultural validity, with very few bespoke, child‐specific measures identified.

**What's relevant?**

- Culturally inappropriate assessment risks misdiagnosis, poor data quality, and ineffective policy and care.
- There is an urgent need for First Nations‐led, culturally grounded, and psychometrically robust tools to support accurate assessment and improved outcomes.

## INTRODUCTION

Aboriginal and Torres Strait Islander people (referred to as First Nations people from hereafter) experience poorer health and wellbeing than the general population, with numerous studies demonstrating the negative impact of colonisation (Australian Institute of Health and Wellbeing [AIHW], 2009, 2018). However, research into the mental health and wellbeing of First Nations people under 12 years of age remains limited. There is evidence of high rates of psychological distress among First Nations children in Western Australia (WA), as measured by the Western Australian Aboriginal Child Health Survey (WAACHS), which found 26.3% of First Nations children (aged 4–11 years old) experienced serious emotional or behavioural difficulties compared to 16.9% of the general population (Zubrick et al., 2005). In 2009–2013, the national mortality data indicated that for First Nations children aged 1–14 years, intentional self‐harm/suicide was over six times the rate for the general population (Australian Bureau of Statistics [ABS], 2014). Other metrics on mental health such as symptoms and prevalence rates are scarce, with very few epidemiological studies capturing the prevalence of mental health conditions specifically in children, in part, due to paucity of culturally valid assessment tools (Jorm et al., 2012; Saunders et al., 2023).

Culturally valid conceptualisations are essential to the assessment of mental ill‐health (Adams et al., 2014; Balaratnasingam et al., 2015; Swan & Raphael, 1995; Westerman, 2021). Cultural validity is often discussed in reference to determining if a construct developed in one cultural group is applicable, meaningful, and equivalent in another cultural group (Keane et al., 1997; Matsumoto, 2003). The Centre for Best Practice in Aboriginal and Torres Strait Islander Suicide Prevention (CBPATSISP) provides important high‐level definitions of cultural validity, categorising assessments as culturally valid, culturally adapted, or not culturally valid. However, the evaluation of cultural validity has lacked a standardised methodological approach. In practice, cultural validity assessments have often relied on subjective judgement and have not consistently addressed established equivalence domains, including content, linguistic, conceptual, scale, technical, and normative equivalence (Flaherty et al., 1988).

Previous systematic reviews have identified that few studies use culturally validated assessment tools to assess mental ill‐health in First Nations adults (Aji, et al., 2025; Black et al., 2018; Castro et al., 2025; Jorm et al., 2012; LeGrande et al., 2017; Newtown et al., 2015). Using assessment tools designed for the general population, as opposed to culturally valid tools, has a number of limitations. For example, one limitation is the use of biomedical conceptualisations of mental ill‐health which are often not aligned with First Nations perspectives, such as the model of SEWB preferred by many (Gee et al., 2014; Saunders et al., 2023). Further, the use of culturally inappropriate assessments can lead to misdiagnoses, which impact on prevalence data which is used to identify funding priorities and wellbeing policy recommendations. It also negatively influences individual treatment outcomes and the cultural safety of services (Balaratnasingam & Janca, 2019; Black et al., 2015; Ralph & Ryan, 2017). Additionally, generic assessments often fail to measure strengths or culturally specific determinants central to SEWB, resulting in deficit‐focused approaches that are culturally misaligned and unable to capture the factors that underpin resilience, wellbeing, and healing for First Nations children (Fogarty et al., 2018; Gee et al., 2014; Le Grande et al., 2017; O’Gradey Lee et al., 2025; Saunders et al., 2023). This limitation has important implications for data sovereignty, as the use of culturally inappropriate measures risks producing data that misrepresents First Nations experiences and priorities (Snijder, 2013).

Given there are very few bespoke culturally valid tools for First Nations people, the use of generic assessments, often without consideration for First Nations ontologies and epistemologies has become standard practice in research. This is alarming given the implications of culturally inappropriate assessment. Although researchers have attempted to adapt generic assessments to improve their cultural validity for First Nations people, there are no assessment tools that enable the evaluation of a measure's cultural validity relevant to First Nations people. This gap in existing methodologies led to the development of a new tool to assess cultural validity: The First Nations Cultural Validity Assessment Tool (FN‐CVAT; O’Gradey‐Lee et al., 2025). The FN‐CVAT is the first tool to assess the cultural validity of assessment tools prioritising First Nations perspectives in addition to assessing other key psychometric properties providing a framework to assess cultural validity in assessment tools.

Although childhood is a critical development period for mental health prevention and intervention (Aji, et al., 2025; Bertie et al., 2026; Francis et al., 2025; Saunders et al., 2023), there is currently no systematic review on tools and their cultural validity utilising an identified framework such as the FN‐CVAT for First Nations children aged 4–12. This gap is significant, given one third of the First Nations population in Australia are under the age of 13 (ABS, 2021). Globally, 50% of mental illnesses onset before the age of 14 (Kessler et al., 2005), and in Australia approximately 13.6% of children aged 4–11 years old experience a mental health condition (Lawrence et al., 2015). The lack of quality research into this development period presents a significant gap in the field and raises several key questions: (1) How is SEWB and mental ill‐health assessed for First Nations children aged 4–12 in research and clinical practice? (2) What bespoke assessment tools are culturally validated for First Nations children? (3) What generic assessment tools are culturally validated, and how have they been validated for use? This review aims to assess the properties of assessment tools in relation to their cultural validity for First Nations children and provide recommendations on the assessment of SEWB and mental health for First Nations children aged 4–12.

## METHOD

This review was conducted in accordance with the Preferred Reporting Items for Systematic Reviews and Meta‐Analyses (PRISMA; Page et al., 2021) and prospectively registered with PROSPERO (registry number: CRD42024542866). This review was conducted through the following three sequential stages. Stage 1: Systematic literature search of mental health and SEWB assessment tools which in children from 4 to 12 years old. Stage 2: Evaluation of the cultural validity of the assessment tools that assess SEWB or mental health using the FN‐CVAT (O’Gradey‐Lee et al., 2025). Stage 3: Recommendations for assessing mental health and SEWB using assessment tools. N.B The terms ‘assessment tool’ or ‘tool’ are used to describe any assessment such as a measure, questionnaire or interview.

### Literature search strategy

**FIGURE 1:**
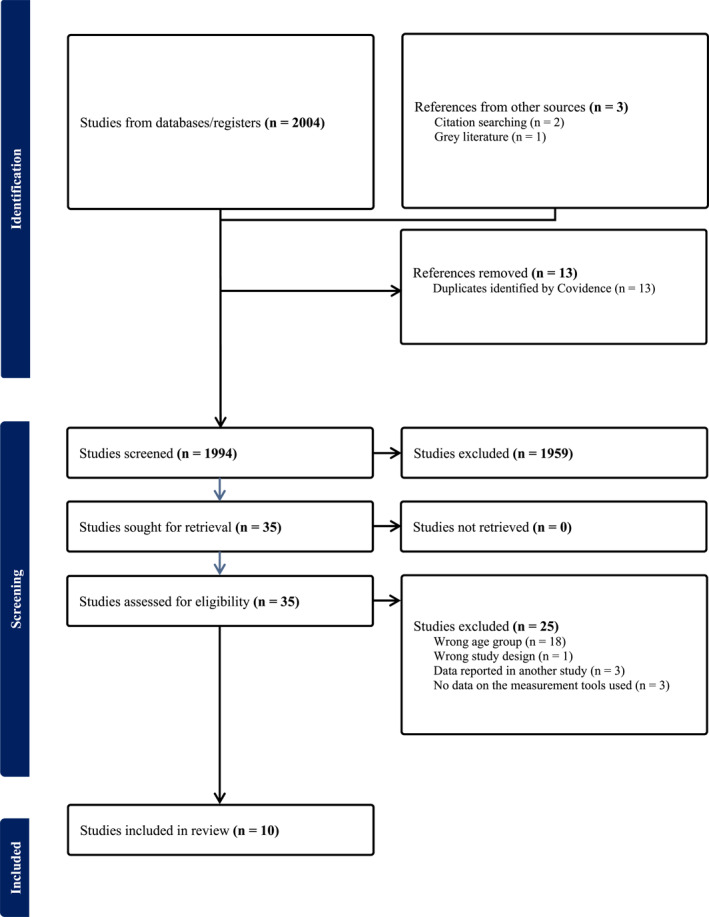
PRISMA flow diagram.

The literature search strategy and Preferred Reporting Items of Systematic Reviews and Meta‐Analyses PRISMA are outlined in Figure 1. The five data bases searched were Web of Science, PubMed, PsycInfo, Informit and CINAHL. The search terms are reported in Supporting Information S1; Appendix S1.

All literature was included, specifically, peer‐reviewed articles and grey literature (i.e., government reports). Retrieved results were imported into Zotero and transferred to Covidence, where duplicates were deleted (*n* = 13). In addition to the search terms, relevant papers found in the reference lists of articles identified in the search and government reports were manually added to Covidence and screened independently for eligibility. Title and abstract screening and full‐text review were conducted using Covidence by two independent reviewers (MO, EM). MO reviewed 100% of abstracts and full texts, while EM reviewed 100% of abstracts and 54.5% of full texts. The agreement rate for abstract screening prior to discussion was *κ* = 0.56, and agreement was perfect for the full‐text review (*κ* = 1).

### Inclusion and exclusion criteria

Inclusion criteria:

1. Written in English language.
2. Articles published between 1980 and 2025.
3. Studies focussing on Aboriginal or Torres Strait Islander youth populations.
4. Participants were aged between 4 and 12 years, with the mean age of the population being <12 or separate analyses were conducted on the age group of 4–12 if part of a larger study.
5. Studies that included the assessment and measurement of mental health or SEWB among First Nations children; using both qualitative and/or quantitative methods, including measurement instruments (self, parent, or teacher report) or clinical interviews for assessment, screening or diagnosis, in research or clinical settings. For studies including Indigenous and non‐Indigenous Australians, mental health outcomes were required to be reported separately for First Nations children.

Social and emotional wellbeing assessments were defined as:

- An assessment that considers: connection to body and behaviours, connection to mind and emotions, connection to family and kinship, connection to community, connection to culture, connection to Country and connection to spirit, spirituality and ancestors the key domains of the SEWB model.
- A paper which describes measuring SEWB outcomes by the authors.

Mental health assessments were defined as

- Assessments on symptoms of mental health conditions, distress or general mental health outcomes.
- A paper which describes measuring mental health outcomes by the authors.

Exclusion:

1. Global focus (i.e., studies do not focus on Aboriginal or Torres Strait Islander people in Australia).
2. Mean age of less than 4 years.
3. Mean age of more than 12 years.
4. Does not focus on SEWB/mental health.
5. Does not include information on the ways mental health was measured (e.g., psychometric measure, interviews, etc.).
6. Systematic/Scoping Review.
7. Protocol papers.
8. Data set was used in a study already included in the review to avoid duplicates.

### Assessing the cultural validity

Assessment tools were classified into three broad categories based on the CBPATSISP definitions: (i) culturally bespoke (ii) culturally adapted, and (iii) generic, neither culturally adapted nor validated (CBPATSISP, 2022). A culturally bespoke assessment is defined as one developed specifically for First Nations people and designed and developed by First Nations clinical and/or cultural experts and empirically validated. Culturally adapted was defined as a pre‐existing tool developed for the general population that was adapted for use with First Nations people and empirically validated. Generic was defined as a tool developed for the general population which has not been studied within or adapted to the First Nations population.

After assessment tools were classified into the CBPATSISP categories, the cultural validity of the assessment tools used within the studies was assessed using a newly developed tool, The FN‐CVAT (O’Gradey‐Lee et al., 2025). The FN‐CVAT includes 14 questions across three overarching themes: psychometric properties, cultural psychometric properties, and cultural competency of staff. The score determines whether the assessment tool meets the criteria. Some criteria are allocated either zero or one point. Others are allocated between zero and two points. Criteria which are scored from zero to two contain benchmarks that determine the allocation of points. To determine the cultural validity of an assessment tool a total score is calculated by adding the 14 criterion scores together, with total scores ranging from 0 to 15. Higher scores indicate greater cultural validity. The FN‐CVAT was designed and developed by First Nations researchers, piloted with researchers, and guided by First Nations research values in addition to commonly used psychometric frameworks for measure development. MO applied the FN‐CVAT to each assessment tool utilised within the studies. Cultural validity is a contextually specific feature of an assessment and is dynamic. To determine cultural validity, the assessment cannot be viewed in isolation; it is assessed in reference to the context of the study, that is, the population of the study, administrators of the assessment, and any further psychometric development and/or validation. Indigenous culture is rooted in relationships and is best understood within its unique local context which is why each assessment is examined within the context of the unique research environment. Each study was analysed in reference to the cultural validity data available at the time of publication. For example, if a tool was utilised in 2018, information on validation or psychometric analyses were only considered if they occurred in or before 2018. This was done to highlight the cultural validity of the tool at the time of use.

Formal assessment of risk of bias in individual studies was not undertaken, as the review focussed on evaluating the cultural validity of assessment tools rather than the internal validity of study designs, or intervention effects.

### Data extraction

Data were extracted from the full texts of studies by MO into a table using Covidence. The descriptive domains extracted for each publication included: authorship, year, age range of participants, constructs measured, symptom period, and reporter. Following the descriptive extraction, data were extracted on the cultural validity properties of each assessment tool used in the respective studies. Data were extracted on assessment tool characteristics and cultural adaptation status. Tools were classified using CBSPATSISP definitions, and FN‐CVAT cultural validity ratings were recorded as reported. No data transformation or statistical conversion was required.

## RESULTS

The results of the search strategy are presented in Figure 1, in accordance with the PRISMA guidelines (Page et al., 2021). We initially retrieved 2007 papers. After the removal of duplicate papers and abstract and titles screening, 35 texts remained which were evaluated for full‐text review which resulted in the inclusion of 10 studies, all of which were included in the narrative synthesis. From the 10 publications, a total of 11 distinct assessment tools were identified. Given that cultural validity is contextually embedded, the cultural validity of an assessment is assessed within the context of each respective study. Thus, cultural validity data are presented for these 11 assessment tools, used in 16 different contexts (Note: one measure ‐ the Strengths and Difficulties Questionnaire (SDQ) ‐ was utilised in six separate studies). As part of the synthesis, assessment tools were classified, using the CBSPATSISP definitions, as bespoke, culturally adapted or generic, based on descriptions of tool development and use within each publication. The review identified three assessment tools which were bespoke, five which were culturally adapted and four which were generic. Assessments such as the SDQ appear in both generic and culturally adapted categories because in some cases the generic tool underwent cultural adaptations. The publications are summarised in Table 1. Cultural validity ratings from the FN‐CVAT are presented in Table 2. Table 3 provides further details and recommendations on the most commonly used screening questionnaires.

**TABLE 1:** Summary of assessment tools and their properties identified in the review.

Author year of publication	Title	Type of study	Population of focus	Age range	Measures used	Construct/s measured	Informant	Time period	Cultural validity (FNCVAT)
Blunden & Chervin (2010)	Sleep, performance and behaviour in Australian indigenous and non‐indigenous children: An exploratory comparison	Case control	First nations specific	7–11.1, *M* = 8.8	CBCL, SDSC	Behavioural sleep problems	Parent	Current symptoms	Poor
FaHCSIA (2013)	Footprints in time: the longitudinal study of indigenous children: Report from wave 4	Cross‐sequential design	First nations specific	0.5–11 B cohort: *M* = 4 K cohort *M* = 7	SDQ	Emotional development, culture and community connection	Parent and child	Current symptoms	Unacceptable
Gartland et al. (2024)	The childhood resilience study: Resilience and emotional and behavioural wellbeing experienced by Australian Aboriginal and Torres Strait islander boys and girls aged 5–9 years	Cohort study	First nations specific	5–9, *M* = 6.5	CRQ‐P/C, SDQ	Resilience and wellbeing	Parent	Current symptoms	Acceptable to excellent
Nikolof et al. (2025)	Mapping Aboriginal children's social and emotional wellbeing: Development and validation of a new tool in an Aboriginal cohort study	Prospective cohort study	First nations specific	6–8; *M* = 6.5	Aboriginal Children's SEWB tool/SDQ	SEWB	Parent	Current	Limited to acceptable
O'Brien et al. (2020)	Physical activity and risk of behavioural and mental health disorders in kindergarten children: Analysis of a series of cross‐sectional complete enumeration (census) surveys	Cross‐sectional	General population	4–6 *M* = 5.27	SDQ	Behavioural and mental health disorders and physical activity	Parent	Current symptoms	Poor
Redman‐MacLaren et al. (2017)	Measuring resilience and risk factors for the psychosocial well‐being of Aboriginal and Torres Strait islander boarding school students: Pilot baseline study results	Interrupted time series design	First nations specific	11–12	CYRM‐28, K5	Resilience and well‐being	Child	Current symptoms	Limited
Reid et al. (2022)	Integrating cultural considerations and developmental screening into an Australian first nations child health check	Cross‐sectional	First nations specific	0.58–17.26; *M* = 5.90	Share and care check, RNDA	Functional concerns and cultural connections	Parent	Current symptoms	Limited
Vance et al. (2022)	Key demographic and mental disorder diagnostic differences between Australian first nations and non‐first nations clinic‐referred children and adolescents assessed in a culturally appropriate and safe way	Prevalence study	First nations specific	6–16, *M* = 11	K‐SADS‐PL, the Rutter and Graham interview schedule	DSM diagnoses	Clinician and parent	Current symptoms	Limited
Williamson et al. (2016)	What are the factors associated with good mental health among Aboriginal children in urban New South Wales, Australia? Phase I findings from the study of environment on Aboriginal resilience and child health (SEARCH)	Cohort study	First nations specific	4–19; *M* = 9	SDQ	Emotional or behavioural problems	Parent	Current symptoms	Acceptable
Zubrick et al. (2005)	The Western Australian Aboriginal child health survey: The social and emotional wellbeing of Aboriginal children and young people	Cross‐sectional	First nations specific	0–17	SDQ	Emotional and behavioural difficulties	Parent	Past symptoms	Limited

Abbreviations: CBCL, Child Behaviour Checklist; CRQ‐P/C, Child Resilience Questionnaire; CYRM‐28, The Child and Youth Resilience Measure; K5, Kesseler Psychological Distress Scale‐5; K‐SADS‐PL, The Kiddie Schedule for Affective Disorders and Schizophrenia Present and Lifetime Version; RNDA, Rapid Neurodevelopmental Assessment; SDQ, Strengths and Difficulties Questionnaire; SDSC, The Sleep Disturbance Scale for Children.

**TABLE 2:** FN‐CVAT Scores of all Assessment Tools used to Measure SWEB within the Review.

Measure	Validated for use with first nations people	Validated in the population of interest	Psychometric data available in the general community	Sensitivity to change available in the general community	Acceptable psychometric properties in population of interest	Available normative data	Measure designed for first nations people	Designed by cultural experts	Adapted for first nations people	Available in indigenous language	Assessment of first nations derived construct	Evidence of staff cultural competency	First nation researcher	Ethics obtained from a first nations committee	Total
Bespoke															
Aboriginal Children's SEWB tool (Nikolof et al., 2025)	0	0	1	0	1	0	2	1	1	0	1	1	1	1	10 acceptable
CRQ‐P/C (Gartland et al., 2024)	1	1	1	0	1	0	2	1	1	0	0	1	1	1	12 excellent
Share the care check (Reid et al., 2022)	0	0	0	0	0	0	2	1	1	0	0	1	1	1	7 limited
Adapted															
CYRM‐28 (Redman‐MacLaren et al., 2017)	0	0	1	1	0	0	0	0	1	0	1	1	1	1	7 limited
K‐SADS‐P/L (Vance et al., 2022)	0	0	1	0	0	0	0	0	1	0	0	1	1	1	5 limited
K5 (Redman‐MacLaren et al., 2017)	1	0	1	0	0	0	0	0	0	0	0	1	1	1	5 limited
SDQ (Gartland et al., 2024)	1	0	1	1	0	0	0	0	1	0	0	1	1	1	7 limited
SDQ (FaHCSIA, 2013)	1	1	1	1	1	0	0	0	1	0	0	1	1	1	9 acceptable
SDQ (Nikolof et al., 2025)	1	0	1	1	0	0	0	0	1	0	0	1	1	1	7 limited
SDQ (Williamson et al., 2014)	1	1	1	1	1	0	0	0	1	0	0	1	1	1	9 acceptable
SDQ (Zubrick et al., 2005)	0	0	1	1	1	0	0	0	1	0	0	1	1	1	7 limited
Generic															
CBCL (Blunden & Chervin, 2010)	0	0	1	1	0	0	0	0	0	0	0	1	0	0	3 poor
RNDA (Reid et al., 2022)	0	0	1	0	0	1	0	0	0	0	0	1	1	1	5 limited
SDCS (Blunden & Chervin, 2010)	0	0	1	0	0	0	0	0	0	0	0	1	0	0	2 poor
RGIS (Vance et al., 2022)	0	0	1	0	0	0	0	0	1	0	0	1	1	1	5 limited
SDQ (O’Brien et al., 2020)	1	0	1	1	0	0	0	0	0	0	0	0	0	0	3 poor

Abbreviations: CBCL, Child Behaviour Checklist; CRQ‐P/C, Child Resilience Questionnaire; CYRM‐28, The Child and Youth Resilience Measure; K5, Kesseler Psychological Distress Scale‐5; K‐SADS‐PL, The Kiddie Schedule for Affective Disorders and Schizophrenia Present and Lifetime Version; RGIS, The Rutter and Graham Interview Schedule; RNDA, Rapid Neurodevelopmental Assessment; SDQ, Strengths and Difficulties Questionnaire; SDSC, The Sleep Disturbance Scale for Children.

**TABLE 3:** Summary of screening questionnaires used to measure mental health or sweb for first nations children.

Measure	Construct	Informant	Age range	Items	Subscale(s)	Response scale	First nations population it has been studied in	Recommendations
Aboriginal Children's SEWB tool	SEWB	Parent/carer	6–8	80	Body, mind/Emotions, family, community, culture & country	0–5 (Varied)	South Australia	A co‐designed SEWB tool developed by first nations people. The tool is comprehensive in its assessment of SEWB. Strong psychometric properties observed in the large initial development study and tool was developed in line with cultural best practices. Further research required to confirm validity in a follow up study and in other communities. The tool shows great promise for use with first nations children
CBCL	Behavioural problems	Parent/carer, child, teacher	1.5–18	110 113	Anxious/depressed, depressed, Somatic complaints, social problems, Thought problems, attention problems, rule‐breaking behaviour & Aggressive behaviour	0–2 (absent‐ occurs often)	N/A	Psychometric validation research required into the cultural validity of the measure
CRQ	Resilience	Parent/carer, child self‐report	5–12	43	Individual (positive self/future, managing emotions), family (connectedness, basic needs, guidance), school (teacher support, engagement, friends) & culture (connectedness, language)	0–4 (not at all—All of the time)	South Australia	Culturally validated for use with first nations children in South Australia. Further validation studies are required to determine the validity of the measure for other communities; however, the measure shows promise to be applicable across communities as it has been co‐designed with first nations people
CYRM	Resilience	Parent/carer, child, ‘person most knowledgeable	5–23	28	Individual, relational, contextual	1–5 or 1–3 (none of the time‐all of the time)	N/A	A social ecological approach to the model design makes this measure a sound option for first nations children as it is recommended by the authors to adapt to the community you are researching Validation of the measure for first nations adolescents has been conducted and found that the model did not fit first nations adolescents, however, it fit within the dynamic conceptualisation of resilience for first nations contexts (Langham et al., 2018). Further research is required
K5	Psychological distress	Child	16+	5	N/A	1–5 (never ‐all the time)	N/A for children	The measure should not be used with children under the age of 16
SDQ	Emotional and behavioural problems	Parent/carer, child, teacher	2–18	25	Emotional problems, conduct problems, hyperactivity/inattention, peer relationships & prosocial	0–2 (not true‐ certainly true)	Urban and regional NSW, across Western Australia, urban, regional and remote areas across Australia	Use the SDQ with caution and only utilise the total difficulties score due to concerns on the internal consistency of subscales and inapplicability. There are concerns over the reliability and validity of subscales demonstrated in large national scale studies and qualitative research from community. If using the measure adaptions are recommended to improve the cultural validity relevant to the community in which the research is being conducted The SDQ has the strongest psychometric properties in urban NSW examined in the SEARCH cohort
SDSC	Sleep disturbance and disorders	Parent	6–15	26	Initiating/maintaining sleep, sleep breathing disorders, disorders of arousal, sleep‐wake disorders, disorders of excessive somnolence & sleep hyperidrosis	0–4 (never‐ Always)	N/A	Psychometric validation research required into the cultural validity of the measure

Abbreviations: CBCL, Child Behaviour Checklist; CRQ‐P/C, Child Resilience Questionnaire; CYRM‐28, The Child and Youth Resilience Measure; K5, Kesseler Psychological Distress Scale‐5; SDQ, Strengths and Difficulties Questionnaire; SDSC, The Sleep Disturbance Scale for Children.

### Generic tools

Four studies used five generic tools: two interviews and three screening questionnaires. The interviews included Rutter and Graham Interview Schedule (RGIS; Rutter & Graham, 1968), and the Rapid Neurodevelopmental Assessment (RNDA; Khan et al., 2010). The screening questionnaires included the Child Behaviour Checklist (CBCL; Achenbach & Rescorla, 2001), the Sleep Disturbance Scale for Children (SDSC; Bruni et al., 1996), and the Strengths and Difficulties Questionnaire (SDQ; Goodman, 2001).

#### The child behaviour checklist

The Child Behaviour Checklist (CBCL; Achenbach & Rescorla, 2001) assesses internalising and externalising problems in children and adolescents. For children aged 1.5–5.5 years, there is a parent report measure, and for children 6–18 years, there are parent report, teacher report, and self‐report measures. The CBCL consists of 110–113 items where respondents rate the frequency of behaviours on a three‐point Likert scale (*absent*, *occurs sometimes*, *occurs often*). It includes eight syndrome subscales and a total score. The raw scores are converted into t‐scores based on the child's age and gender, a t‐score ≤59 indicates non‐clinical symptoms, a t‐score between 60 and 64 indicates that the child is at risk for problem behaviours, and a t‐score ≥65 indicates clinical levels of symptoms. The CBCL is available for purchase. It demonstrates high internal consistency across all its subscales, excellent test re‐test reliability and is sensitive to change (Achenbach & Rescorla, 2001; Cohen et al., 2004; Swenson et al., 2010). Although the CBCL has been tested in diverse cultures, clinical cut‐offs are not uniform (Tackett & Awong, 2008). No information could be found on the use of the CBCL within First Nations children in Australia. The CBCL (Achenbach & Rescorla, 2001) was used by Blunden and Chervin's (2010) study on sleep and behaviour problems. When the FN‐CVAT was applied to this study, it scored three out of 15 placing it in the poor range for cultural validity.

#### The sleep disturbance scale for children

The Sleep Disturbance Scale for Children (SDSC; Bruni et al., 1996) is a screening tool for sleep disturbances and specific sleep disorders in children aged six to 15, used in both clinical practice and research. The SDSC is a 26‐item parent report measure where parents rate the frequency of behaviours on a four‐point Likert scale (*never*, *occasionally*, *sometimes*, *always*). It includes six subscales and a total score. Higher scores indicate more acute sleep disturbances (Bruni et al., 1996). The SDSC has standardised scoring by converting the raw score into a t‐score: a t‐score of <50 places a child in the ‘normal’ range, a *t*‐score between 50 and 70 places a child in the ‘borderline’ range and a t‐score of >70 placing a child in the ‘clinically significant’ range (Bruni et al., 1996; Mancini & Pearcy, 2021). The SDSC has fair to good internal consistency, good test re‐test reliability, excellent diagnostic accuracy of 0.91 and adequate sensitivity and specificity (Bruni et al., 1996; Mancini & Pearcy, 2021). The SDSC has been used in a variety of cultures and disorders (Lewandowski et al., 2011). No information could be found on the use of the SDSC with First Nations children in Australia, nor have other peer‐reviewed studies utilised the measure for First Nations children between the ages of 4–12. In Blunden and Chervin's (2010) study, the SDSC scored two out of 15 on the FN‐CVAT, placing it in the poor range for cultural validity.

#### The Rutter and Graham interview schedule

The Rutter and Graham Interview Schedule (RGIS) (Rutter & Graham, 1968) is a semi‐structured clinical interview that assesses symptom severity, distress and impairment rated to psychiatric disorders. It has a child and parent interview, where the clinician rates each area on a three‐point Likert‐type scale (*absent*, *mild*, *severe*). The Rutter and Graham Interview Schedule has good test–retest reliability, internal consistency and concurrent validity (Rutter & Graham, 1968). The Rutter and Graham Interview Schedule is not commonly used in research now due to the development of new assessments such as the Diagnostic Interview Schedule for Children Version and Anxiety Disorder Interview Schedule for Children (Silverman & Albano, 1996). Limited information was available on psychometric properties of the measure, nor on its applicability across cultures. When the FN‐CVAT was applied to RGIS in Vance and colleagues (2022) study, it scored five, placing it in the poor range for cultural validity.

#### The rapid neurodevelopmental assessment

The Rapid Neurodevelopmental Assessment (RNDA; Khan et al., 2010) is a 27‐item assessment tool administered by practitioners that is designed to detect functional status and impairment across multiple neurodevelopmental domains in children aged 0–16. It was developed in Bangladesh for low‐resource settings and can be delivered by non‐specialists. It is now being tested for use in First Nations rural and remote communities (Campbell et al., 2025; Reid et al., 2022). The RNDA assesses nine domains gross and fine motor, vision, hearing, speech, cognition, behaviour, self‐care, and seizures. There is emerging evidence for its use in First Nations communities, Campbell and colleagues (2025) assessed the eight items within the behavioural domain. They found that the RNDA has good convergent validity as evidenced by significant Point‐biserial correlations between the RNDA and the Behavioural Assessment Schedule for Children‐3 (BASC‐3) and most items had acceptable accuracy as evidenced by comparisons of indicators between the BASC‐3 and RNDA (Campbell et al., 2025). The RNDA hyperactivity, attention problems, externalising problems, and behaviour symptoms index demonstrated high sensitivity of 82% or greater (Campbell et al., 2025). However, the remaining items demonstrated mixed sensitivity with items falling between 14% and 71%, and specificity ranged from 29% to 88% (Campbell et al., 2025). The lowest item for sensitivity was anxiety indicating that this item was not worded in a culturally appropriate way (Campbell et al., 2025). When the FN‐CVAT was applied to the RNDA within the Share the Care study (Reid et al., 2022), it scored five, placing it in the poor range. The ongoing validation studies of the RNDA show promise for its validity and reliability in First Nations communities (Campbell et al., 2025).

#### The strengths and difficulties questionnaire

The SDQ^1^ (SDQ; Goodman, 2001) measures psychopathology and prosocial behaviour in children through five dimensions; emotional symptoms, hyperactivity‐inattention, conduct problems, peer problems, and prosocial behaviour. It can be completed by an informant such as a parent, carer or teacher or the child can complete a self‐report from aged seven and above. It is a brief measure of 25 items in which respondents rate the extent to which items are true for themselves or their child on a three‐point Likert scale (*not true*, *somewhat true*, *certainly true*). A score can be derived from each subscale or by summing the subscale scores for a total score. Higher scores indicate greater difficulties. The SDQ is freely available, there is normative data for Australian children, but not for First Nations children (Mellor, 2005). The SDQ is one of the most frequently used measures of child mental health globally and has been translated into more than 80 languages (Stolk et al., 2017). There is mixed evidence on the reliability of the SDQ, with various studies deeming the internal consistency of the SDQ between satisfactory and strong (Goodman, 2001; Yao et al., 2009), with subscales ranging from poor to acceptable (Hawes & Dadds, 2004; Muris et al., 2004; Yao et al., 2009). The SDQ has shown moderate test‐retest reliability, good concurrent, convergent, and discriminant validity (Ludh et al., 2008; Muris et al., 2004; Yao et al., 2009).

Despite the popularity of the measure, there is a growing body of evidence that demonstrates shortcomings in its applicability across cultures including for First Nations children in Australia (Chau et al., 2023; Garrido et al., 2020; Santiago et al., 2021; Williamson et al., 2010, 2014). For example, qualitative studies conducted by Williamson et al. (2010); Zubrick et al. (2005), supported the acceptability of the measure, however, concerns around the validity and reliability of subscales have been raised particularly the peer subscale. The peer subscale demonstrated poor internal consistency (Williamson et al., 2014). Qualitative studies support this with First Nations families and health workers, reporting that the subscale is poorly aligned with Indigenous views of relationships, and neglected to consider the role of the wider kinship system crucial to wellbeing for First Nations children (Williamson et al., 2010). Concerns with the hyperactivity scale were also reported by participants who felt the questions were not necessarily indicative of a clinical problem rather they were better explained by social factors such as parenting (Williamson et al., 2010).

Given the multiplicity of the SDQ across contexts, the SDQ will be discussed in greater detail within the context of each study. The SDQ was used in the Kindergarten Health Check (KHC; O’Brien et al., 2020), an annual cross‐sectional survey of all children in the Australian Capital Territory in their first year of full‐time primary education. In the sample 2.3% identified as Indigenous (O’Brien et al., 2020). There were no adaptations made to the SDQ within this research. When the FN‐CVAT was applied to the SDQ in the context of the KHC it scored three out of 15 placing it in the poor range for cultural validity. Adapted versions of the SDQ were utilised in four other studies (see below).

### Culturally adapted

Seven studies used four unique culturally adapted assessment tools. These include the SDQ (Goodman, 2001), The Kiddie Schedule for Affective Disorders and Schizophrenia Present and Lifetime Version (Kaufman et al., 2000), The Child and Youth Resilience Measure (CYRM‐28) (CYRM; Ungar & Liebenberg, 2011) and Kesseler Psychological Distress Scale‐5 (K‐5; Kessler & Mroczek, 1992).

#### Strengths and difficulties questionnaire

Five studies used an adapted version of the SDQ for their respective studies. Each study took a different adaptation to the tool. Four studies were large cohort studies: The Longitudinal study of Indigenous children (LSIC) also known as the Footprints in Time study, the WAACHS, the Study of Environment on Aboriginal Resilience and Child Health (SEARCH) and the Aboriginal Families Study (AFS). The LSIC is a national study of First Nations children, it is an accelerated cross‐sequential design involving two cohorts: one aged from 6 months to 2 years (B cohort) and one aged from 3 years to 6 months to 5 years (K cohort) with data collected annually for 5 years (Department of Families, Housing, Community Services and Indigenous Affairs [FaHCSIA], 2013). The WAACHS was a cross‐sectional study into First Nations children's wellbeing and development within WA, the largest study of its kind in WA (Zubrick et al., 2005). The AFS is a prospective cohort study investigating wellbeing in Aboriginal children living in South Australia. The SEARCH is a cohort study of First Nations children aged 0–17 years in urban New South Wales (NSW) examining the causes of health and illness through a strengths‐based lens. It is the largest cohort study of urban Aboriginal children ever conducted (The SEARCH Investigators, 2010).

The LSIC, WAACHS and SEARCH studies all administered the SDQ as an interview with an Aboriginal researcher present. The SDQ was assessed and determined to be valid for the SEARCH cohort and demonstrated acceptable construct validity (Williamson et al., 2016). When the FN‐CVAT was applied to the SEARCH (Williamson et al., 2016), the SDQ scored nine out of 15, placing it within the acceptable range.

In the LSIC study in later waves, some of the questions were translated into traditional languages, but this did not undergo linguistic validation. A psychometric validation of the LSIC by Santiago and colleagues (2021) found poor internal consistency across subscales, and they were unable to replicate the factor structure of the SDQ utilising the five, four or three factor model indicating poor validity. They did not conduct any other validity or inter‐rater reliability analyses. These results indicate the SDQ is not a valid measure of emotional and behavioural difficulties in First Nations children and adolescents and using the subscales to screen is not valid (Santiago et al., 2021). Chau and colleagues (2023) examined the psychometric properties within the LSIC and found similar results to previous studies. Namely, mixed internal consistency with most scales having poor reliability (Cronbach alphas fell between α.24‐ *α*. 67) particularly within the parent report, however, the total difficulties score demonstrated acceptable internal consistency *α* > 0.70 (Chau et al., 2023). When the FN‐CVAT was applied to the LSIC (FaHCSIA, 2013), it scored nine out of 15, placing it within the acceptable range.

For the WAACHS study the carers were shown a prompt card with the responses in addition to minor word changes to the questions and response categories (i.e., changing somewhat true to sometimes). The reliability of the SDQ within the WAACHS (Zubrick et al., 2005) was robust for all subscales including the total score except for the peer subscale (Zubrick & Lawrence, 2006). Validity of the measure was strongest when applied to the total score, thus the authors concluded that the total score provides a reasonable measure of wellbeing for First Nations children (Zubrick & Lawrence, 2006). When the FN‐CVAT was applied to the WAACHS (Zubrick et al., 2005) it scored seven out of 15, placing it in the limited range. The cultural validity of the SDQ in the WAACHs is lower than the other cohort studies despite similar procedures because at the time of the WAACHs the SDQ had not been widely used in First Nations focussed research as it has since. This highlights how cultural validity is dynamic and contextually specific not only to place, but to time as well.

Both Nikolof et al. (2025) and Gartland et al. (2024) adapted the interpretation of the SDQ scoring by framing responses as positive SEWB and negative SEWB utilising a subscale of high mental health competence to examine respondents who are flourishing as opposed to examining difficulties this strengths‐based approach is more aligned with Indigenous research values (Gartland et al., 2023; O’Connor et al., 2022). However, it is important to note, this subscale of mental health competence has not undergone any psychometric validation which limits the ability to truly evaluate the cultural validity. The SDQ within the context of Gartland and colleagues (2024) and Nikolof and colleagues (2025) study scored seven on the FN‐CVAT, placing it in the limited cultural validity range.

#### The kiddie schedule for affective disorders and schizophrenia present and lifetime version

The Kiddie Schedule for Affective Disorders and Schizophrenia Present and Lifetime Version (K‐SADS‐PL; Kaufman et al., 2000) is a semi‐structured diagnostic interview based on Diagnostic Statistic Manual (DSM‐IV) criteria that assesses psychiatric disorders in children and adolescents aged 6–18. The K‐SDAS‐PL can be administered to the child and the parent; summary ratings are obtained in the interview and a diagnosis can be made utilising this and clinical judgement. There is also growing evidence this can be used for pre‐school aged children (Birmaher et al., 2009). The K‐SDAS‐PL demonstrates good to excellent internal consistency, excellent inter‐rater reliability, excellent agreement between separate raters, fair to excellent test re‐test reliability and good to excellent concurrent and convergent validity (Birmaher et al., 2009; Kaufman et al., 2000; Kim et al., 2004).

The K‐SADS‐PL has been studied across numerous cultures and translated into over 30 languages and demonstrated commensurate psychometric properties to the original (Brasil & Bordin, 2010; Marques et al., 2022, 2022rðarson et al., 2020). The use of the K‐SADS‐PL with First Nations children is not empirically documented, outside the scope of this paper there has been some research using the K‐SADS‐PL within prison populations including First Nations adolescents (Indig et al., 2011). However, there is minimal data available on the psychometric properties of the measure (Indig et al., 2011). Vance and Colleagues (2022) note the limitations of the measure for First Nations populations and made adaptations to the interpretation of the measure through consultation with First Nations mental health staff identifying culturally valid impairing patterns of symptoms. Additionally, First Nations people conducted the assessments improving the cultural validity of the measure overall. The K‐SADS‐PL was used in Vance and Colleagues (2022) study and scored five on the FN‐CVAT placing it in the limited range for cultural validity.

#### The child and youth resilience measure

The Child and Youth Resilience Measure (CYRM; Ungar & Liebenberg, 2011) is a 28‐item screening tool to assess resilience in children and young people aged 5–23 years old. The CYRM was developed in collaboration with 11 countries selected to represent the diverse social context and risks in young people's lives (Ungar & Liebenberg, 2011). The measure assesses individual, peer, family and community resources and can be completed as a self‐report or an informant report. Items are rated on either a three‐point or five‐point Likert scale, with higher scores indicating higher resilience. The CYRM takes a social ecological approach acknowledging that the context in which the child is in influences their resilience. There is normative data available, however, the authors recommend contrasting high and low scorers within the sample, additionally the authors recommend adapting the measure to suit the research or clinical population and provide guidance on how to do this within the manual (Ungar & Liebenberg, 2011). The CYRM has strong psychometric properties including acceptable internal consistency, inter‐rater reliability, convergent and concurrent validity (Daigneault et al., 2013; Ungar, 2006).

Psychometric validation work has been undertaken on the measure for First Nations adolescents, which is however outside of the scope of the present review of child measures (Langham et al., 2018). In the Resilience study pilot data (Redman‐Maclaren et al., 2017) minor adaptations in wording and response scales were made to reflect students lived experience. When the FN‐CVAT was applied to the CYRM in the context of Redman‐Maclaren and colleagues (2017) study it scored seven, placing it in the limited range for cultural validity.

#### The Kessler psychological distress scale‐5

The Kessler Psychological Distress Scale‐5 (K‐5; Kessler & Mroczek, 1992) is a five‐item measure used to assess general psychological health and distress. Originally adapted from the K‐10, the K5 is widely used to assess distress in adult First Nations populations including in large government surveys and has shown promise as a tool to measure distress (McNamara et al., 2014). The K‐5 has been adapted for First Nations populations through minor word changes to the questions such as without hope instead of hopeless. The K‐5 is commonly used with First Nations children and youth; however, no studies have examined the cultural appropriateness of the tool for children (Black Dog Institute., 2022; Jorm et al., 2012). The K‐5 has shown promising psychometric properties within adult populations and work has been conducted to culturally adapt the K‐5 for First Nations adults known as the MK‐K5 (Brinckley et al., 2021; Jorm et al., 2012; McNamara et al., 2014). The FN‐CVAT was applied to the K‐5 in the context of the Resilience study pilot data (Redman‐Maclaren et al., 2017) and scored three placing it in the limited range for cultural validity.

### Culturally bespoke tools

Three studies utilised three unique tools specifically developed for research or service delivery for First Nations children. These measures included a questionnaire within the Share the Care check (Reid et al., 2022), the Child Resilience Questionnaire (CRQ‐P/C; Gartland et al., 2022a) and the Aboriginal Children's SEWB Tool (Nikolof et al., 2025).

#### Share and care check

The Share and Care check is a co‐designed Children's health check led by Aboriginal practitioners from a remote Aboriginal Community Controlled Health organisation (Reid et al., 2022). The health check was designed to take a holistic perspective to children's health and wellbeing by assessing health and cultural connectedness in a culturally appropriate way (Reid et al., 2022). The check was completed by First Nations practitioners as this was deemed as crucial by the community as First Nations workers were identified as having an enhanced understanding of the community's needs (Reid et al., 2022). The Share and Care check has 13 components utilising a combination of generic assessment items discussed above and specifically designed items such as connection to country. The Share and Care check provides an example of a culturally appropriate approach to healthcare. The check received positive qualitative feedback from participants, however, there is no additional data on the psychometrics of the questionnaire. This is a common problem within the field wherein measures are designed for First Nations people, but no psychometric validation is conducted on the measure which raises the question of the validity of the measure. The Share and Care check scored seven out of 15 on the FN‐CVAT, placing it in the limited range for cultural validity.

#### Child resilience questionnaire

Child Resilience Questionnaire (CRQ‐P/C/S; Gartland et al., 2022a; Gartland et al., 2022b) is a 43‐item questionnaire that assesses resilience in children. There are three measures, a self‐report measure for children aged 7–12 years old (CRQ‐C), a parent/carer version for children aged 5–12 years old (CRQ‐P) and a school staff version for children aged 5–12 (CRQ‐S). The CRQ was co‐designed with Aboriginal communities across South Australia and refugee communities. The measure was designed specifically for children in Australia focussing on First Nations children and refugees who have experienced a high level of exposure to early life adversity and ongoing exposure to trauma and discrimination (Gartland et al., 2022a). The authors utilised participatory action research to co‐design the measure, utilised a strengths‐based approach to the questionnaire and also had First Nations leadership and governance embedded throughout the measure development process (Gartland et al., 2022a, 2022b, 2024). The questionnaire has 10 scales including: self (positive self, positive future, managing emotions); family (connectedness, guidance, basic needs); school (teacher support, engagement, friends); and culture (connectedness, language) with response options on a Likert scale between 0 and 4 (*not often*, *sometimes*, *most of the time*, *and all of the time*) with the option of a pictogram of a glass for response items (Gartland et al., 2022a). Higher scores indicate greater resilience. The questionnaire has good construct validity and criterion validity, as well as excellent reliability with internal consistency for the total score (Gartland et al., 2022a). It also had acceptable to excellent internal consistency for the subscales except for the ‘basic needs’ subscale within the family domain which was poor *α* = 0.61 (Gartland et al., 2022a). The CRQ was utilised in Gartland and colleagues (2024) study on resilience in middle childhood and scored 12 on the FN‐CVAT placing it in the excellent range for cultural validity.

#### Aboriginal children's social and emotional wellbeing tool

The Aboriginal Children's SEWB (Nikolof et al., 2025) is an 80‐item carer‐report measure that assesses SEWB in Aboriginal children. The tool is the first Aboriginal‐led co‐designed tool to map SEWB based on the SEWB model by Gee and colleagues (2014). Items were retrospectively drafted from the AFS study which was developed with community including items from the CRQ and grouped into indicators by an Aboriginal researcher. Item drafting was an iterative process with strong community consultation throughout the process with the final draft measure piloted in a group of 222 participants. Items were organised into 29 indicators representing children's SEWB across six domains: Body, Mind and Emotions, Family and Kinship, Community, Culture and Country, and Spirit and Spirituality. The tool scores domains as positive or less positive based on the number of positive indicators, domain scores are totalled to a create a total SEWB score which classifies children into high, medium and low SEWB. The tool underwent a comprehensive psychometric validation. To reflect the diversity among the items, a Cronbach of ≥0.3 was deemed as appropriate for internal consistency. Five domains exhibited acceptable internal consistency; however, the community domain did not meet requirements, following consultation with cultural governance groups the items in the domain were amended and internal consistency was met. Overall, the measure demonstrated good internal consistency *α* = 0.69 and criterion validity with scores from the tool compared to results on the SDQ. The tool's strength lies in its development which consisted of rigorous community consultation and the fact the tool takes a strengths‐based lens. Further psychometric testing is required, and is planned by the authors to expand the measure for 5–17 year olds. On the FN‐CVAT the Aboriginal Children's SEWB Tool scored 10, falling in the acceptable range. It is important to note that the tool received a lower score because it is newly developed and has not yet been assessed across two studies, meaning it does not currently meet the criteria required to be considered validated in the FN‐CVAT. There is strong preliminary evidence of this tools cultural validity due to the rigorous development process in line with First Nations ontologies and epistemologies.

## DISCUSSION

This review is the first to assess the cultural validity of mental health or wellbeing assessment tools for First Nations children. The findings provide the first detailed analysis of cultural validity and highlight the widespread use of culturally inappropriate assessment tools. The review identified that the majority of research uses generic or adapted tools, out of the 11 tools identified, only three were bespoke (a tool designed specifically for First Nations people). Using the FN‐CVAT, the first quality assessment tool designed to assess cultural validity of tools in First Nations communities, assessment tools identified in the review scored between two to 12. Across the 15 assessments of cultural validity, over three quarters were within the poor or limited range for cultural validity. Only one tool CRQ‐P/C (Gartland et al., 2022a, 2022b) was identified as having excellent cultural validity in the context of the specific research study. The CRQ measures resilience, which is only one component of SEWB. One bespoke tool was identified the Aboriginal Children's SEWB Tool (Nikolof et al., 2025) which scored in the acceptable range. No bespoke mental health tools were identified and only two studies used a culturally adapted assessment of mental health that scored within the acceptable range for cultural validity the SDQ (FaHCSIA, 2013; Williamson et al., 2014).

The limited availability and use of culturally validated tools and poor cultural validity within the measurement of SEWB and mental ill‐health for First Nations children is alarming but not surprising. There is increasing recognition that First Nations people conceptualise mental ill‐health through a different lens than the dominant psychiatric model commonly used within research and clinical practice (Fogarty et al., 2018; LeGrande et al., 2017). Despite this, it has remained common practice to employ assessments which do not take into consideration Indigenous ways of knowing, being and doing (Black Dog Institute, 2022; Castro et al., 2025; LeGrande et al., 2017; Snijder, 2013). This review adds to the growing body of evidence that there is a lack of appropriate assessment tools for First Nations people (Black et al., 2018; Castro et al., 2025; Chau et al., 2023; Jorm et al., 2012; Legrande et al., 2017; Newtown et al., 2015). The findings align with previous reviews (Black et al., 2018; Newtown et al., 2015) that within the assessment of SEWB and mental health the majority of assessment tools focus on deficits or psychological problems and lack adequate psychometric evaluation. This review provides the first evaluation of cultural validity using an identified First Nations framework which has a sole focus on First Nations children.

### Use of culturally valid tools

A significant gap in the literature is the psychometric evaluation of bespoke assessment tools, which is a crucial step to ensure their overall reliability and validity (LeGrande et al., 2017). Psychometric information was difficult to ascertain across several tools assessed in the review. More transparency is needed in the reporting of measures psychometric properties to be able to critically evaluate the research and design future measures. The development of culturally valid assessment tools is imperative to support the SEWB of First Nations children.

Two of the bespoke assessment tools, the CRQ (Gartland et al., 2022a) and the Aboriginal Children's SEWB Tool (Nikolof et al., 2025) had available psychometric evaluation data and scored in the acceptable to excellent range. The recent development of bespoke SEWB and mental health assessments for First Nations people marks a positive step towards addressing the limitations of generic and adapted tools. These include the CRQ, the SEWB Tool discussed above, Strong Souls, and the Westerman Aboriginal Symptom Checklist (WASC‐Y/WASC‐A; Adams et al., 2014). Both the Strong Souls and WASC‐Y were developed for adults and adolescents aged 13 and above not children highlighting the need for child‐specific measure development. The SEWB Tool (Nikolof et al., 2025) was developed to meet this gap and provides positive future directions for the assessment of SEWB in children.

### Use of culturally adapted tools

Measure adaptations often included minor changes to the wording of the questions, with the exception of one study that adapted the interpretation of the individual's scoring to be strengths‐focused, as opposed to deficit‐focused (Gartland et al., 2024; Nikolof et al., 2025). This is an example of working in line with First Nations health frameworks, by taking a strengths‐based approach. Whilst it is favourable to utilise a tool specifically designed for First Nations people, we acknowledge this is not always possible within research or clinical practice.

There are potential advantages to the utilisation of culturally adapted generic tools that have been adapted for their cultural appropriateness, as these have the potential to allow for comparisons across groups when adequate normative data is available for different cultural groups (Zubrick & Lawrence, 2006). It is important to note though, that adaptations should be led by First Nations people and with consultations from the community of interest. This is particularly useful in large government health surveys as seen in the LSIC and the Longitudinal Study of Australian Children. When a generic or adapted tool has been utilised, it is imperative that the limitations of the research are clearly noted. Within the adult literature there are good examples of culturally adapted generic tools for the assessment of mental ill‐health (LeGrande et al., 2017). The PHQ9 (Brown et al., 2013) provides a promising example of the cultural adaptation process of a generic measure to suit First Nations communities (LeGrande et al., 2017). However, adapting a tool is a difficult process which requires strong cultural guidance and First Nations leadership to ensure its cultural validity and appropriateness. Adapting tools is complicated by the non‐homogenous nature of First Nations communities in regard to cultural practices, cultural knowledge and conceptualisations and language (Marmion et al., 2014).

### Use of generic tools

The inadequacy of generic assessment tools for First Nations people has been documented considerably in recent years (Chau et al., 2023; Santiago et al., 2021; Williamson et al., 2014). Despite this, these tools continue to be used in research for First Nations children, as there are few culturally valid alternatives highlighting the urgent need for the development and use of culturally appropriate and valid tools for research and clinical practice. The SDQ was the most frequently used measure within the literature. The SDQ has mixed results for its cultural validity (Chau et al., 2023; Santiago et al., 2021; Williamson et al., 2014; Zubrick et al., 2005). As a result, many researchers are positioned against the use of the SDQ for First Nations children (Chau et al., 2023; Santiago et al., 2021). Although the SDQ demonstrated the strongest psychometric properties namely construct validity, convergent validity and reliability in relation to the carer report for urban and regional areas of NSW, Williamson and colleagues (2014) advocate that adaptations should be made to the questions and not to interpret the peer subscale, due to concerns around its validity for First Nations children. Subsequently, the SDQ should not be the gold standard measure used in research for First Nations children, especially without adaptations to the questions and interpretation. If the SDQ is used, the subscales should not be interpreted, particularly the peer subscale. Instead, the Total difficulties score is preferred, noting its limitations of cultural validity reported alongside the interpretation of results.

### Implications

Assessment is used to quantify and understand the needs of an individual or a community. Data gathered from assessments of mental health and SEWB, play a key role in enhancing our understanding of the needs of an individual and/or a community (Jennings et al., 2018). If the assessment tool is not culturally valid, then the data gathered is not culturally appropriate. The fact that assessment tools are not culturally validated for First Nations children but are used frequently exemplifies data ignorance, defined as the production and use of data that fail to accurately represent the lived experiences, priorities, and contexts of the populations they are intended to reflect (Harnett & Featherstone, 2020). This is a critical concern, as these data shape future research, policy directions, treatment programs and funding allocation (Jennings et al., 2018). The use of culturally inappropriate and invalid assessment tools has significant cascading effects for First Nations people. These include poorer treatment outcomes, the perpetuation of harmful research practices, ineffective and at times harmful governmental policies and inappropriate allocation of funds into ‘problem’ areas or ineffective clinical care (Bond & Singh, 2020; Ralph & Ryan, 2017).

### Strengths and limitations

One of the limitations of this review is that we did not engage with local communities to discover what measures are being used at a community level. We relied heavily on searches of databases and, despite including grey literature, it is likely that we missed tools. The use of database searches privileges Western academic models of research over community driven resources and assessments. Despite this, the review is the first to focus on First Nations children in Australia and the first to use an assessment tool to determine cultural validity and provide quantitative and qualitative data on their psychometric properties in relation to cultural validity. Previous reviews have only provided narrative reviews of measurement tools or focussed on adolescent or adult populations (Black et al., 2018). The review did not assess other areas of study quality and only focussed on the cultural validity of the measurement tool itself, whilst this is an important area of focus, it did narrow the focus of the review which limited the overall appraisal of the research.

Additionally, the tool used to assess cultural validity, the FN‐CVAT (O’Gradey‐Lee et al., 2025), has its own limitations and strengths. The FN‐CVAT combines Indigenous ways of knowing, being and doing with biomedical and psychological constructs important to psychometric validation and assessment. This was the first tool developed to assess cultural validity utilising First Nations values and frameworks and has only undergone pilot testing under the supervision of cultural experts. The FN‐CVAT prioritises First Nations knowledge in line with established ethical guidelines and principles hence the additional points for First Nations led design and categorisation of measures into three categories acknowledging their classification. However, whilst every effort has been made to include tools specifically designed for First Nations people, psychometric evaluations in peer‐reviewed publications are scarce, thus, this has led to one First Nations‐led bespoke measure receiving a ‘limited’ cultural validity score, identifying the need for future development.

Additionally, while some studies reported community consultation in the development of bespoke tools, there was inconsistent reporting regarding whether culturally adapted tools involved meaningful community consultation. This limited our ability to fully evaluate the cultural validity of adapted measures and highlights a broader issue of insufficient transparency in reporting.

### Recommendations

Based on the cultural validity framework and review findings, we provide the following recommendations for the assessment of First Nations children's mental health and SEWB:

1. As a gold standard, researchers and clinicians should ideally utilise bespoke tools specifically designed for First Nations children that have been validated for use in research and/or clinical practice. This also includes ensuring that the chosen tool is culturally valid for the intended population.
2. When reliable and valid bespoke tools are not available, culturally adapted tools should be used, bearing in mind the limitations. This may also involve the researcher or clinician making appropriate adaptations of the tool. Cultural adaptations should be made under the guidance of First Nations people such as First Nations researchers and cultural governance groups whose lived experience matches the population of interest. The process of adapting the measure should be clearly reported within the publication so it can be replicated by future research.
3. We recommend against the use of generic tools, given the significant impact on the quality of the research and its implications. When a generic tool has been utilised, its applicability and validity should be approached with caution. This should be clearly noted within the research limitations.
4. Apply the FN‐CVAT to potential assessment to support clinicians and researchers in their decision‐making. Future research should examine the impact of FN‐CVAT application to decision‐making to further establish its effectiveness.
5. More investment is required in the development of culturally appropriate measures led by First Nations researchers and evaluation of the measures' cultural validity including aspects such as internal consistency, construct validity, content validity and inter‐rater reliability in the population of interest.
6. When reporting on research conducted with First Nations children, it is essential to include the psychometric properties of the assessment relevant to the study sample. This transparency will encourage increased psychometric evaluation of measures, ensuring their reliability and validity across cultures. The lack of cultural perspectives when using Western assessment tools with individuals from non‐Western cultures represents a significant gap in the literature. This gap greatly impacts the ability of researchers and clinicians to determine the relevance of these measure for individuals outside Western culture. For example, providing data on internal consistency, inter‐rater reliability, convergent validity, and divergent validity from the First Nations sample in addition to the main objectives of the study, would be beneficial.
7. Researchers and clinicians are encouraged to view mental health through the SEWB framework and to adopt a strengths‐based approach where applicable.
8. Journals should require authors to report on the cultural validity of assessment tools used with First Nations populations, including detailing any adaptations made and evidence supporting their use. This will promote transparency, improve methodological rigour, and support the development of a stronger evidence base.
9. Funding bodies should prioritise and resource the development and validation of culturally appropriate assessment tools led by First Nations researchers and communities. Sustained investment in this area is critical to advancing culturally valid measurement and supporting Indigenous data sovereignty.

## CONCLUSION

Assessment tools typically used to assess SEWB and mental health in First Nations children in research demonstrate poor cultural validity. The majority of publications included in this systematic review utilised an adapted version of a generic assessment; the SDQ. Only two assessments designed to assess SEWB for First Nations children had undergone psychometric evaluation. As demonstrated by the poor cultural validity scores, it is apparent that generic tools often fail to conceptualise and assess SEWB from Indigenous perspectives, however, are frequently used despite their poor cultural validity and subsequent limitations. Culturally valid tools for First Nations children are scarce and of the culturally adapted tools for mental health they predominantly demonstrated limited cultural validity. The lack of appropriate and culturally valid assessment tools has significant impacts on the SEWB and mental health of First Nations children and consequently on research, clinical care and policies aimed at improving SEWB for First Nations children. The development of culturally valid measurement tools led by First Nations people like the Aboriginal Children's SEWB Tool is imperative to enhance the SEWB and mental health of First Nations children.

## AUTHOR CONTRIBUTIONS

**Maddison O’Gradey‐Lee**: Conceptualization; data curation; formal analysis; writing—original draft; methodology; investigation; project administration; writing—review and editing. **Emma A. McDermott**: Writing—review and editing; project administration. **Lizel‐Antoinette Bertie**: Writing—review and editing; methodology; data curation. **Clinton Schultz**: Conceptualization; supervision; writing—review and editing. **Jennifer L. Hudson**: Conceptualization; investigation; funding acquisition; methodology; writing—review and editing; supervision; resources.

## CONFLICT OF INTEREST STATEMENT

The authors declare no conflicts of interest.

## ETHICAL CONSIDERATIONS

This study is a systematic review of existing published literature and did not involve primary data collection or direct contact with human participants. Therefore, ethical approval was not required, consistent with institutional and international guidelines for research using secondary data.

## Supporting information

Supporting Information S1

## Data Availability

All data analysed in this study were obtained through a systematic search of publicly available literature in the following electronic databases: Web of Science, PubMed, PsycINFO, Informit, and CINAHL. All data extracted and analysed are included within the manuscript.

## ENDNOTE

^1^
The SDQ appears in both the generic measures and culturally adapted measures section. In one study, it was used in its standard form and in others, a culturally adapted version was used.

## References

[jcv270147-bib-0001] Achenbach, T. M. , & Rescorla, L. A. (2001). Manual for the ASEBA school‐age forms & profiles. University of Vermont, Research Center for Children, Youth, & Families.

[jcv270147-bib-0002] Adams, Y. , Drew, N. , & Walker, R. (2014). Principles of practice in mental health assessment with aboriginal Australians. In P. Dudgeon , H. Milroy , & R. Walker (Eds.), Working together: Aboriginal and torres strait islander mental health and wellbeing principles and practice (pp. 271–288). Commonwealth of Australia.

[jcv270147-bib-0003] Aji, M. , Xu, X. , McDermott, E. A. , Metz, M. , Songco, A. , O’Gradey‐Lee, M. , Lim, C. Y. S. , Sicouri, G. , Parrish, L. , & Hudson, J. L. (2025). Measures of sleep‐Related fears in children: A systematic review of psychometric properties using COSMIN. Clinical Child and Family Psychology Review, 28(2), 439–457. 10.1007/s10567-025-00526-6 40397330 PMC12162706

[jcv270147-bib-0004] Australian Institute of Health and Welfare (AIHW) . (2009). Measuring the social and emotional wellbeing of Aboriginal and Torres strait islander peoples. AIHW. Retrieved from https://www.aihw.gov.au/reports/indigenous‐australians/measuring‐the‐social‐and‐emotional‐wellbeing/summary

[jcv270147-bib-0005] Australian Bureau of Statistics . (2014). Causes of death, Australia 2014. ABS website. Retrieved from http://www.abs.gov.au/ausstats/abs.nsf/Lookup/by%20Subject/3303.0~2014~Main%20Features~Intentional%20self‐harm%20by%20Age~10051

[jcv270147-bib-0006] Australian Bureau of Statistics . (2021). Aboriginal and Torres Strait Islander people: Census. ABS. Retrieved from https://www.abs.gov.au/statistics/people/aboriginal‐and‐torres‐strait‐islander‐peoples/aboriginal‐and‐torres‐strait‐islander‐people‐census/2021

[jcv270147-bib-0007] Australian Institute of Health and Wellbeing (AIHW) . (2018). Aboriginal and Torres Strait Islander adolescent and youth health and wellbeing 2018. AIHW. Retrieved from https://www.aihw.gov.au/reports/indigenous‐australians/atsi‐adolescent‐youth‐health‐wellbeing‐2018/contents/summary

[jcv270147-bib-0008] Balaratnasingam, S. , Anderson, L. , Janca, A. , & Lee, J. (2015). Towards culturally appropriate assessment of Aboriginal and Torres strait islander social and emotional well‐being. Australasian Psychiatry, 23(6), 626–629. 10.1177/1039856215608283 26432653

[jcv270147-bib-0009] Balaratnasingam, S. , & Janca, A. (2019). Depression in Indigenous Australians: Getting it right. Medical Journal of Australia, 211(1), 16–17. 10.5694/mja2.50230 31168796

[jcv270147-bib-0010] Bertie, L. A. , Mackinnon, A. , McDermott, E. A. , O’Gradey‐Lee, M. , Kikas, K. , Tomlinson, M. , & Hudson, J. L. (2026). Moving beyond moderation to identify differential treatment effects of cognitive behavioural therapy for childhood anxiety: A systematic review. Clinical Child and Family Psychology Review, 1–25. 10.1007/s10567-026-00564-8 41790339 PMC13562111

[jcv270147-bib-0011] Birmaher, B. , Ehmann, M. , Axelson, D. A. , Goldstein, B. I. , Monk, K. , Kalas, C. , Kupfer, D. , Gill, M. K. , Leibenluft, E. , Bridge, J. , Guyer, A. , Egger, H. L. , & Brent, D. A. (2009). Schedule for affective disorders and schizophrenia for school‐age children (K‐SADS‐PL) for the assessment of preschool children—A preliminary psychometric study. Journal of Psychiatric Research, 43(7), 680–686. 10.1016/j.jpsychires.2008.10.003 19000625 PMC2736874

[jcv270147-bib-0012] Black, E. B. , Ranmuthugala, G. , Kondalsamy‐Chennakesavan, S. , Toombs, M. R. , Nicholson, G. C. , & Kisely, S. (2015). A systematic review: Identifying the prevalence rates of psychiatric disorder in Australia’s indigenous populations. Australian and New Zealand Journal of Psychiatry, 49(5), 412–429. 10.1177/0004867415569802 25690747

[jcv270147-bib-0013] Black, E. B. , Toombs, M. R. , & Kisely, S. (2018). The cultural validity of diagnostic psychiatric measures for Indigenous Australians. Australian Psychologist, 53(5), 383–393. 10.1111/ap.12335

[jcv270147-bib-0014] Black Dog Institute . (2022). Turning the tide on depression: A vision that starts with Australia’s youth Sydney. Black Dog Institute. Retrieved from https://www.blackdoginstitute.org.au/wp‐content/uploads/2022/10/Youth‐Depression‐Report_Full.pdf

[jcv270147-bib-0015] Blunden, S. , & Chervin, R. D. (2010). Sleep, performance and behaviour in Australian indigenous and non‐indigenous children: An exploratory comparison. Journal of Paediatrics and Child Health, 46(1–2), 10–16. 10.1111/j.1440-1754.2009.01610.x 19943868

[jcv270147-bib-0016] Bond, C. J. , & Singh, D. (2020). More than a refresh required for closing the gap of Indigenous health inequality. Medical Journal of Australia, 212(5), 198. 10.5694/mja2.50498 32030749

[jcv270147-bib-0017] Brasil, H. H. , & Bordin, I. A. (2010). Convergent validity of K‐SADS‐PL by comparison with CBCL in a Portuguese speaking outpatient population. BMC Psychiatry, 10(1), 83. 10.1186/1471-244X-10-83 20955616 PMC2984471

[jcv270147-bib-0018] Brinckley, M.‐M. , Calabria, B. , Walker, J. , Thurber, K. A. , & Lovett, R. (2021). Reliability, validity, and clinical utility of a culturally modified Kessler scale (MK‐K5) in the Aboriginal and Torres Strait Islander population. BMC Public Health, 21(1), 1111. 10.1186/s12889-021-11138-4 34112127 PMC8194217

[jcv270147-bib-0019] Brown, A. D. , Mentha, R. , Rowley, K. G. , Skinner, T. , Davy, C. , & O’Dea, K. (2013). Depression in Aboriginal men in central Australia: Adaptation of the patient health questionnaire 9. BMC Psychiatry, 13(1), 271. 10.1186/1471-244X-13-271 24139186 PMC3816593

[jcv270147-bib-0020] Bruni, O. , Ottaviano, S. , Guidetti, V. , Romoli, M. , Innocenzi, M. , Cortesi, F. , & Giannotti, F. (1996). The sleep disturbance scale for children (SDSC) construct ion and validation of an instrument to evaluate sleep disturbances in childhood and adolescence. Journal of Sleep Research, 5(4), 251–261. 10.1111/j.1365-2869.1996.00251.x 9065877

[jcv270147-bib-0021] Campbell, T. , Shanley, D. C. , Page, M. , McDonald, T. , Zimmer‐Gembeck, M. , Hess, M. , Watney, J. , & Hawkins, E. (2025). Psychometric properties of the rapid neurodevelopmental assessment in detecting social‐emotional problems during routine child developmental monitoring in primary healthcare. Review, 26(1), 106. 10.1186/s12875-025-02807-z PMC11987427 40217187

[jcv270147-bib-0022] Castro, M. J. , Gall, Z. , Gall, A. , Smith, H. , & Kunaratnam, K. (2025). Validated and culturally specific screening tools and early response programs for the detection and prevention of eating disorders among first nations peoples in Australia: A scoping review. Journal of Eating Disorders, 13(1), 167. 10.1186/s40337-025-01334-7 40760454 PMC12323021

[jcv270147-bib-0023] Centre for Best Practice . (2022). Best practice screening & assessment. Retrieved from https://cbpatsisp.com.au/clearing‐house/best‐practice‐screening‐assessment/

[jcv270147-bib-0024] Chau, T. , Tiego, J. , Brown, L. , Coghill, D. , Jobson, L. , Montgomery, A. , Murrup‐Stewart, C. , Sciberras, E. , Silk, T. J. , Spencer‐Smith, M. , Stefanac, N. , Sullivan, D. P. , & Bellgrove, M. A. (2023). Against the use of the strengths and difficulties questionnaire for Aboriginal and Torres Strait Islander children aged 2–15 years. Australian and New Zealand Journal of Psychiatry, 57(10), 1343–1358. 10.1177/00048674231161504 36974891 PMC10517593

[jcv270147-bib-0025] Cohen, J. A. , Deblinger, E. , Mannarino, A. P. , & Steer, R. A. (2004). A multisite, randomized controlled trial for children with sexual abuse–related PTSD symptoms. Journal of the American Academy of Child & Adolescent Psychiatry, 43(4), 393–402. 10.1097/00004583-200404000-00005 15187799 PMC1201422

[jcv270147-bib-0026] Daigneault, I. , Dion, J. , Hébert, M. , McDuff, P. , & Collin‐Vézina, D. (2013). Psychometric properties of the child and youth resilience measure (CYRM‐28) among samples of French‐Canadian youth. Child Abuse & Neglect, 37(2–3), 160–171. 10.1016/j.chiabu.2012.06.004 23260113

[jcv270147-bib-0027] Department of Families, Housing, Community Services and Indigenous Affairs (FaHCSIA) . (2013). Footprints in time: The longitudinal study of Indigenous children: Report from wave 4. Retrieved from https://www.dss.gov.au/system/files/documents/2024‐10/footprints_in_time_wave4.pdf

[jcv270147-bib-0028] Flaherty, J. A. , Gaviria, F. M. , Pathak, D. , Mitchell, T. , Wintrob, R. , Richman, J. A. , & Birz, S. (1988). Developing instruments for cross‐cultural psychiatric research. The Journal of Nervous and Mental Disease, 176(5), 260–263. 10.1097/00005053-198805000-00001 3367140

[jcv270147-bib-0029] Fogarty, W. , Lovell, M. , Langenberg, J. , & Heron, M. J. (2018). Deficit discourse and strengths‐based approaches. Changing the Narrative of Aboriginal and Torres Strait Islander Health and Wellbeing Melbourne: The Lowitja Institute.

[jcv270147-bib-0030] Francis, D. A. , Sicouri, G. , Songco, A. , Lim, C. Y. , McDermott, E. A. , Werner‐Seidler, A. , Reily, N. , Allsop, A. , Aji, M. , Thingbak, A. , O’Gradey‐Lee, M. , Chen, W. , & Hudson, J. L. (2025). Identifying and supporting school‐aged children and young people with anxiety and depressive disorders: The evidence and next steps. Australian Psychologist, 60(6), 471–483. 10.1080/00050067.2025.2567672

[jcv270147-bib-0031] Garrido, L. E. , Barrada, J. R. , Aguasvivas, J. A. , Martínez‐Molina, A. , Arias, V. B. , Golino, H. F. , Legaz, E. , Ferrís, G. , & Rojo‐Moreno, L. (2020). Is small still beautiful for the strengths and difficulties questionnaire? Novel findings using exploratory structural equation modeling. Assessment, 27(6), 1349–1367. 10.1177/1073191118780461 29911418

[jcv270147-bib-0032] Gartland, D. , Nikolof, A. , Glover, K. , Leane, C. , Cahir, P. , Hameed, M. , & Brown, S. J. (2023). Patterns of health and health service use in a prospective cohort of Aboriginal and torres Strait islander children aged 5–9 years living in urban, regional and remote areas of South Australia. International Journal of Environmental Research and Public Health, 20(12), 6172. 10.3390/ijerph20126172 37372759 PMC10298277

[jcv270147-bib-0033] Gartland, D. , Nikolof, A. , Mensah, F. , Gee, G. , Glover, K. , Leane, C. , Carter, H. , & Brown, S. J. (2024). The childhood resilience study: Resilience and emotional and behavioural wellbeing experienced by Australian Aboriginal and Torres Strait Islander boys and girls aged 5–9 years. PLoS One, 19(4 April), e0301620. 10.1371/journal.pone.0301620 38626131 PMC11020770

[jcv270147-bib-0034] Gartland, D. , Riggs, E. , Giallo, R. , Glover, K. , Stowe, M. , Mongta, S. , Weetra, D. , & Brown, S. J. (2022a). Development and validation of a multidimensional, culturally and socially inclusive child resilience questionnaire (parent/caregiver report) to measure factors that support resilience: A community‐based participatory research and psychometric testing study in Australia. BMJ Open, 12(6), e061129. 10.1136/bmjopen-2022-061129 PMC9214413 35725263

[jcv270147-bib-0035] Gartland, D. , Riggs, E. , Giallo, R. , Glover, K. , Stowe, M. , Mongta, S. , Weetra, D. , & Brown, S. J. (2022b). Development of a multidimensional culturally and socially inclusive measure of factors that support resilience: Child resilience questionnaire‐child report (CRQ‐C)—a community‐based participatory research and psychometric testing study in Australia. BMJ Open, 12(9), e060229. 10.1136/bmjopen-2021-060229 PMC9486312 36113941

[jcv270147-bib-0036] Gee, G. , Dudgeon, P. , Schultz, C. , Hart, A. , & Kelly, K. (2014). Aboriginal and torres Strait islander social and emotional wellbeing. In P. Dudgeon , H. Milroy , & R. Walker (Eds.), Working together: Aboriginal and torres Strait islander mental health and wellbeing principles and practice (2nd ed., pp. 55–58). Commonwealth Government of Australia. Retrieved from https://www.telethonkids.org.au/globalassets/media/documents/aboriginal‐health/working‐together‐second‐edition/wt‐part‐1‐chapt‐4‐final.pdf

[jcv270147-bib-0037] Goodman, R. (2001). Psychometric properties of the strengths and difficulties questionnaire. Journal of the American Academy of Child & Adolescent Psychiatry, 40(11), 1337–1345. 10.1097/00004583-200111000-00015 11699809

[jcv270147-bib-0038] Harnett, P. H. , & Featherstone, G. (2020). The role of decision making in the over‐representation of Aboriginal and Torres Strait Islander children in the Australian child protection system. Children and Youth Services Review, 113, 105019. 10.1016/j.childyouth.2020.105019

[jcv270147-bib-0039] Hawes, D. J. , & Dadds, M. R. (2004). Australian data and psychometric properties of the strengths and difficulties questionnaire. Australian and New Zealand Journal of Psychiatry, 38(8), 644–651. 10.1080/j.1440-1614.2004.01427.x 15298588

[jcv270147-bib-0040] Indig, D. , Vecchiato, C. , Haysom, L. , Beilby, R. , Carter, J. , Champion, U. , Gaskin, C. , Heller, E. , Kumar, S. , Mamone, N. , Muir, P. , van den Dolder, P. , & Whitton, G. (2011). 2009 NSW young people in custody health survey: Full report. Justice Health and Juvenile Justice.

[jcv270147-bib-0041] Jennings, L. L. , David‐Chavez, D. M. , Martinez, A. , Lone Bear Rodriguez, D. , & Rainie, S. (2018). Indigenous data sovereignty: How scientists and researchers can empower Indigenous data governance. 2018, PA43C‐1376. AGU Fall Meeting Abstracts.

[jcv270147-bib-0042] Jorm, A. F. , Bourchier, S. J. , Cvetkovski, S. , & Stewart, G. (2012). Mental health of Indigenous Australians: A review of findings from community surveys. Medical Journal of Australia, 196(2), 118–121. 10.5694/mja11.10041 22304605

[jcv270147-bib-0043] Kaufman, J. , Birmaher, B. , Brent, D. A. , Ryan, N. D. , & Rao, U. (2000). K‐sads‐pl. Journal of the American Academy of Child & Adolescent Psychiatry, 39(10), 1208. 10.1097/00004583-200010000-00002 11026169

[jcv270147-bib-0044] Keane, T. M. , Kaloupek, D. G. , & Weathers, F. W. (1997). Ethnocultural considerations in the assessment of PTSD. In A. J. Marsella , M. J. Friedman , E. T. Gerrity , & R. M. Scurfield (Eds.), Ethnocultural aspects of posttraumatic stress disorder: Issues, research, and clinical applications (pp. 183–205). American Psychological Association. 10.1037/10555-007

[jcv270147-bib-0045] Kessler, R. , & Mroczek, D. (1992). An update of the development of mental health screening scales for the US national health interview study. Survey Research Center of the Institute for Social Research. University of Michigan.

[jcv270147-bib-0046] Kessler, R. C. , Berglund, P. , Demler, O. , Jin, R. , Merikangas, K. R. , & Walters, E. E. (2005). Lifetime prevalence and age‐of‐onset distributions of DSM‐IV disorders in the national comorbidity survey replication. Archives of General Psychiatry, 62(6), 593–602. 10.1001/archpsyc.62.6.593 15939837

[jcv270147-bib-0047] Khan, N. Z. , Muslima, H. , Begum, D. , Shilpi, A. B. , Akhter, S. , Bilkis, K. , Begum, N. , Parveen, M. , Ferdous, S. , Morshed, R. , Batra, M. , & Darmstadt, G. L. (2010). Validation of rapid neurodevelopmental assessment instrument for under‐two‐year‐old children in Bangladesh. Pediatrics, 125(4), e755–e762. 10.1542/peds.2008-3471 20308214

[jcv270147-bib-0048] Kim, Y. S. , Cheon, K.‐A. , Kim, B.‐N. , Chang, S.‐A. , Yoo, H.‐J. , Kim, J.‐W. , Cho, S.‐C. , Seo, D.‐H. , Bae, M.‐O. , So, Y.‐K. , Noh, J.‐S. , Koh, Y.‐J. , McBurnett, K. , & Leventhal, B. (2004). The reliability and validity of Kiddie‐schedule for affective disorders and schizophrenia‐present and lifetime Version‐ Korean version (K‐SADS‐PL‐K). Yonsei Medical Journal, 45(1), 81–89. 10.3349/ymj.2004.45.1.81 15004873

[jcv270147-bib-0049] Langham, E. , McCalman, J. , Redman‐MacLaren, M. , Hunter, E. , Wenitong, M. , Britton, A. , Rutherford, K. , Saunders, V. , Ungar, M. , & Bainbridge, R. (2018). Validation and factor analysis of the child and youth resilience measure for Indigenous Australian boarding school students. Frontiers in Public Health, 6, 299. 10.3389/fpubh.2018.00299 30406069 PMC6206896

[jcv270147-bib-0050] Lawrence, D. , Hafekost, J. , Johnson, S. E. , Saw, S. , Buckingham, W. J. , Sawyer, M. G. , Ainley, J. , & Zubrick, S. R. (2015). Key findings from the second Australian child and adolescent survey of mental health and wellbeing. Australian and New Zealand Journal of Psychiatry, 50(9), 876–886. 10.1177/0004867415617836 26644606

[jcv270147-bib-0051] Le Grande, M. , Ski, C. F. , Thompson, D. R. , Scuffham, P. , Kularatna, S. , Jackson, A. C. , & Brown, A. (2017). Social and emotional wellbeing assessment instruments for use with Indigenous Australians: A critical review. Social Science & Medicine, 187, 164–173. 10.1016/j.socscimed.2017.06.046 28689090

[jcv270147-bib-0052] Lewandowski, A. S. , Toliver‐Sokol, M. , & Palermo, T. M. (2011). Evidence‐based review of subjective pediatric sleep measures. Journal of Pediatric Psychology, 36(7), 780–793. 10.1093/jpepsy/jsq119 21227912 PMC3146754

[jcv270147-bib-0053] Lundh, L. , Wångby‐Lundh, M. , & Bjärehed, J. (2008). Self‐reported emotional and behavioral problems in Swedish 14 to 15‐year‐old adolescents: A study with the self‐report version of the strengths and difficulties questionnaire. Scandinavian Journal of Psychology, 49(6), 523–532. 10.1111/j.1467-9450.2008.00668.x 18489532

[jcv270147-bib-0054] Mancini, V. O. , & Pearcy, B. T. D. (2021). Sensitivity of the child behaviour checklist sleep items and convergent validity with the sleep disorders scale for children in a paediatric ADHD sample. Sleep Medicine, 3, 100033. 10.1016/j.sleepx.2021.100033 PMC8041104 33870180

[jcv270147-bib-0055] Marmion, D. , Obata, K. , & Troy, J. (2014). Community, identity, wellbeing: The report of the second national Indigenous languages survey. Australian Institute of Aboriginal and Torres Strait Islander Studies.

[jcv270147-bib-0056] Marques, C. C. , Matos, A. P. , do Céu Salvador, M. , Arnarson, E. Ö. , & Craighead, W. E. (2022). Reliability and validity of the schedule for affective disorders and schizophrenia for school‐age children‐present and lifetime version (K‐SADS‐PL): Portuguese version. Child Psychiatry and Human Development, 53(6), 1119–1128. 10.1007/s10578-021-01196-5 34050391

[jcv270147-bib-0057] Matsumoto, D. (2003). Cross‐cultural research. In S. F. Davis (Ed.), Handbook of research methods in experimental psychology. Blackwell.

[jcv270147-bib-0058] McNamara, B. J. , Banks, E. , Gubhaju, L. , Williamson, A. , Joshy, G. , Raphael, B. , & Eades, S. J. (2014). Measuring psychological distress in older Aboriginal and Torres Strait Islanders Australians: A comparison of the K‐10 and K‐5. Australian & New Zealand Journal of Public Health, 38(6), 567–573. 10.1111/1753-6405.12271 25307151

[jcv270147-bib-0059] Mellor, D. (2005). Normative data for the strengths and difficulties questionnaire in Australia. Australian Psychologist, 40(3), 215–222. 10.1080/00050060500243475

[jcv270147-bib-0060] Muris, P. , Meesters, C. , Eijkelenboom, A. , & Vincken, M. (2004). The self‐report version of the strengths and difficulties questionnaire: Its psychometric properties in 8‐ to 13‐year‐old non‐clinical children. British Journal of Clinical Psychology, 43(4), 437–448. 10.1348/0144665042388982 15530213

[jcv270147-bib-0061] Newton, D. , Day, A. , Gillies, C. , & Fernandez, E. (2015). A review of evidence‐based evaluation of measures for assessing social and emotional well‐being in Indigenous Australians. Australian Psychologist, 50(1), 40–50. 10.1111/ap.12064

[jcv270147-bib-0062] Nikolof, A. , Gartland, D. , Glover, K. , Leane, C. , Carmody, R. , Cater, H. , Brown, S. J. , & Clark, Y. (2025). Mapping Aboriginal children’s social and emotional wellbeing: Development and validation of a new tool in an Aboriginal cohort study. First Nations Health & Wellbeing – The Lowitja Journal, 3(4), 100062. 10.1016/j.fnhli.2025.10006

[jcv270147-bib-0063] Obrien, K. , Agostino, J. , Ciszek, K. , & Douglas, K. A. (2020). Physical activity and risk of behavioural and mental health disorders in kindergarten children: Analysis of a series of cross‐sectional complete enumeration (census) surveys. BMJ Open, 10(3), e034847. 10.1136/bmjopen-2019-034847 PMC7103808 32198302

[jcv270147-bib-0064] O’Connor, M. , Arnup, S. J. , Mensah, F. , Olsson, C. , Goldfeld, S. , Viner, R. M. , & Hope, S. (2022). Natural history of mental health competence from childhood to adolescence. Journal of Epidemiology & Community Health, 76(2), 133–139. 10.1136/jech-2021-216761 34400516 PMC8762025

[jcv270147-bib-0065] O’Gradey‐Lee, M. , Schultz, C. , & Hudson, J. L. (2025). The development of a cultural validity assessment tool for first nations people. Australian and New Zealand Journal of Psychiatry, 60(3), 282–291. 10.1177/00048674251393167 41384575

[jcv270147-bib-0066] Page, M. J. , McKenzie, J. E. , Bossuyt, P. M. , Boutron, I. , Hoffmann, T. C. , Mulrow, C. D. , Shamseer, L. , Tetzlaff, J. M. , Akl, E. A. , Brennan, S. E. , Chou, R. , Glanville, J. , Grimshaw, J. M. , Hróbjartsson, A. , Lalu, M. M. , Li, T. , Loder, E. W. , Mayo‐Wilson, E. , McDonald, S. , & Moher, D. (2021). The PRISMA 2020 statement: An updated guideline for reporting systematic reviews. BMJ, n71, n71. 10.1136/bmj.n71 PMC8005924 33782057

[jcv270147-bib-0067] Ralph, S. , & Ryan, K. (2017). Addressing the mental health gap in working with Indigenous youth: Some considerations for non‐indigenous psychologists working with Indigenous youth. Australian Psychologist, 52(4), 288–298. 10.1111/ap.12287

[jcv270147-bib-0068] Redman‐MacLaren, M. L. , Klieve, H. , Mccalman, J. , Russo, S. , Rutherford, K. , Wenitong, M. , & Bainbridge, R. G. (2017). Measuring resilience and risk factors for the psychosocial well‐being of Aboriginal and torres strait islander boarding school students: Pilot baseline study results. Frontiers in Education, 2. 10.3389/feduc.2017.00005

[jcv270147-bib-0069] Reid, N. , Page, M. , McDonald, T. , Hawkins, E. , Liu, W. , Webster, H. , White, C. , Shelton, D. , Katsikitis, M. , Wood, A. , Draper, B. , Moritz, K. , & Shanley, D. C. (2022). Integrating cultural considerations and developmental screening into an Australian first Nations child health check. Australian Journal of Primary Health, 28(3), 207–214. 10.1071/PY20300 35287792

[jcv270147-bib-0070] Rutter, M. , & Graham, P. (1968). The reliability and validity of the psychiatric assessment of the child: I. Interview with the child. British Journal of Psychiatry, 114(510), 563–579. 10.1192/bjp.114.510.563 5654133

[jcv270147-bib-0071] Santiago, P. H. R. , Manzini Macedo, D. , Haag, D. , Roberts, R. , Smithers, L. , Hedges, J. , & Jamieson, L. (2021). Exploratory graph analysis of the strengths and difficulties questionnaire for Aboriginal and/or torres strait islander children. Frontiers in Psychology, 12, 573825. 10.3389/fpsyg.2021.573825 34484017 PMC8416422

[jcv270147-bib-0072] Saunders, V. , McCalman, J. , Tsey, S. , Askew, D. , Campbell, S. , Jongen, C. , Angelo, C. , Spurling, G. , & Cadet‐James, Y. (2023). Counting what counts: A systematic scoping review of instruments used in primary healthcare services to measure the wellbeing of Indigenous children and youth. BMC Primary Care, 24(1), 51. 10.1186/s12875-023-02001-z 36803458 PMC9936129

[jcv270147-bib-0073] Silverman, W. K. , & Albano, A. M. (1996). The anxiety disorders interview schedule for DSM–IV—Child and parent versions. Psychological Corporation.

[jcv270147-bib-0074] Snijder, M. (2013). Researching right way: Aboriginal and Torres Strait Islander health research ethics – A domestic and. Lowitja Institute. Retrieved from https://policycommons.net/artifacts/2281324/researching‐right‐way/

[jcv270147-bib-0075] Stolk, Y. , Kaplan, I. , & Szwarc, J. (2017). Review of the strengths and difficulties questionnaire translated into languages spoken by children and adolescents of refugee background. International Journal of Methods in Psychiatric Research, 26(4), e1568. 10.1002/mpr.1568 28449279 PMC6877132

[jcv270147-bib-0076] Swan, P. , & Raphael, B. (1995). Ways forward: National consultancy report on Aboriginal and Torres Strait islander mental health. AGPS.

[jcv270147-bib-0077] Swenson, C. C. , Schaeffer, C. M. , Henggeler, S. W. , Faldowski, R. , & Mayhew, A. M. (2010). Multisystemic therapy for child abuse and neglect: A randomized effectiveness trial. Journal of Family Psychology: JFP: journal of the Division of Family Psychology of the American Psychological Association (Division 43), 24(4), 497–507. 10.1037/a0020324 20731496 PMC2928578

[jcv270147-bib-0078] Tackett, J. L. , Awong, T. , Achenbach, T. M. , & Rescorla, L. A. (2008). Multicultural understanding of child and adolescent psychopathology: Implications for mental health assessment. Guilford Press. 2007, ISBN 10:1593853483. Journal of Youth and Adolescence, 37(4), 488–491. 10.1007/s10964-007-9229-8

[jcv270147-bib-0079] The SEARCH Investigators . (2010). The study of environment on Aboriginal resilience and child health (SEARCH): Study protocol. BMC Public Health, 10(1), 287. 10.1186/1471-2458-10-287 20507632 PMC2896939

[jcv270147-bib-0080] Ungar, M. (2006). Resilience across cultures. British Journal of Social Work, 38(2), 218–235. 10.1093/bjsw/bcl343

[jcv270147-bib-0081] Ungar, M. , & Liebenberg, L. (2011). Child and youth resilience measure [Dataset]. PsycTESTS Dataset. 10.1037/t23633-000

[jcv270147-bib-0082] Vance, A. , Winther, J. , McGaw, J. , & White, S. (2022). Key demographic and mental disorder diagnostic differences between Australian first nations and non‐First nations clinic‐referred children and adolescents assessed in a culturally appropriate and safe way. Australian and New Zealand Journal of Psychiatry, 56(11), 1455–1462. 10.1177/00048674211063819 34875892

[jcv270147-bib-0083] Westerman, T. (2021). Culture‐bound syndromes in Aboriginal Australian populations. Clinical Psychologist, 25(1), 19–35. 10.1080/13284207.2020.1843967

[jcv270147-bib-0084] Williamson, A. , D’Este, C. , Clapham, K. , Redman, S. , Manton, T. , Eades, S. , Schuster, L. , & Raphael, B. (2016). What are the factors associated with good mental health among Aboriginal children in urban New South Wales, Australia? Phase I findings from the study of environment on Aboriginal resilience and child Health (SEARCH). BMJ Open, 6(7), e011182. 10.1136/bmjopen-2016-011182 PMC4947800 27381207

[jcv270147-bib-0085] Williamson, A. , Mcelduff, P. , Dadds, M. , D’Este, C. , Redman, S. , Raphael, B. , Daniels, J. , & Eades, S. (2014). The construct validity of the strengths and difficulties questionnaire for Aboriginal children living in urban New South Wales, Australia. Australian Psychologist, 49(3), 163–170. 10.1111/ap.12045

[jcv270147-bib-0086] Williamson, A. , Redman, S. , Dadds, M. , Daniels, J. , D’Este, C. , Raphael, B. , Eades, S. , & Skinner, T. (2010). Acceptability of an emotional and behavioural screening tool for children in Aboriginal community controlled health services in urban NSW. Australian and New Zealand Journal of Psychiatry, 44(10), 894–900. 10.3109/00048674.2010.489505 20932203

[jcv270147-bib-0087] Yao, S. , Zhang, C. , Zhu, X. , Jing, X. , McWhinnie, C. M. , & Abela, J. R. Z. (2009). Measuring adolescent psychopathology: Psychometric properties of the self‐report strengths and difficulties questionnaire in a sample of Chinese adolescents. Journal of Adolescent Health, 45(1), 55–62. 10.1016/j.jadohealth.2008.11.006 19541250

[jcv270147-bib-0088] Zubrick, S. , & Lawrence, D. (2006). Testing the reliability of a measure of Aboriginal children’s mental health: An analysis based on the Western Australian Aboriginal child health survey. Australian Bureau of Statistics.

[jcv270147-bib-0089] Zubrick, S. R. , Silburn, S. R. , Lawrence, D. M. , Mitrou, F. G. , Dalby, R. B. , Blair, E. M. , Griffin, J. , Milroy, H. , De Maio, J. A. , Cox, A. , & Li, J. (2005). The Western Australian Aboriginal child health survey: The social and emotional wellbeing of Aboriginal children and young people. Curtin University of Technology and Telethon Institute for Child Health Research. Retrieved from https://test.telethonkids.org.au/globalassets/media/documents/aboriginal‐health/waachs‐vol2/western_australian_aboriginal_child_health_survey_main_volume.pdf

[jcv270147-bib-0090] Þórðarson, Ó. , Ævarsson, F. M. , Helgadóttir, S. , Lauth, B. , Wessman, I. , Sigurjónsdóttir, S. A. , Smárason, O. , Harðardóttir, H. H. , & Skarphedinsson, G. (2020). Icelandic translation and reliability data on the DSM‐5 version of the schedule for affective disorders and schizophrenia for school‐aged children – Present and lifetime version (K‐SADS‐PL). Nordic Journal of Psychiatry, 74(6), 423–428. 10.1080/08039488.2020.1733660 32134350

