# Supplementary material for: Measurement of social and emotional wellbeing and mental health outcomes in first nations children: A systematic review
Source: JCPP Adv. 2026 Jul 17:e70147. Online ahead of print. doi: 10.1002/jcv2.70147 (PMC13376799; doi:10.1002/jcv2.70147)
Supplement: Supplementary file 1 — Supporting Information S1 [file JCV2-9999-e70147-s001.docx]

**Measurement of social and emotional wellbeing and mental health outcomes in First Nations children: A Systematic Review**

**Supporting Information**

**Appendix S1. Search Terms**

1. Aboriginal or Cultur* or Torres Strait or First Nations or Indigen* and
2. anxiety or wellbeing or Racis* or social and emotional wellbeing or emotion or SEWB or Psychological distress or suicide or grief or trauma or stigma or depression or mental health or Discrimination or Longing for country or sorry cutting or culture-bound
3. Teen* or Adolesc* or child* or young people or youth
4. Assessment or measurement or tool or interview or self-report
